# Comparison short time discharge with long time discharge following uncomplicated percutaneous coronary intervention for Non-ST elevation myocardial infarction patients

**DOI:** 10.1186/s12872-019-1096-1

**Published:** 2019-05-14

**Authors:** Guozhong Wang, Quanming Zhao, Qing Cheng, Xiaoxia Zhang, Lei Tian, Xiaofan Wu

**Affiliations:** Cardiology Department of Beijing Anzhen Hospital, Capital Medical University, Beijing Institute of Heart ,Lung and Blood Vessel Diseases, Chaoyang district AnzhenRoad 2#, Beijing, China

**Keywords:** Coronary artery disease, Non-ST elevation myocardial infarction, Percutaneous coronary intervention, Length of stay, Discharge

## Abstract

**Background:**

The rational length of stay following non-complicated percutaneous coronary intervention (PCI) for Non-ST elevation myocardial infarction (NSTEMI) patients remains controversial. Few studies have examined the impact of early discharge on short-term outcomes in NSTEMI patients, but short-time discharge is not uncommon in real world practice. This study examined the impact of short time discharge following non-complicated PCI on 30-day net adverse clinical events in NSTEMI patients.

**Methods:**

This retrospective study enrolled 1424 consecutive patients with NSTEMI diagnoses who underwent non-complicated PCI. Of these patients, 432 were discharged early (< 24 h), whereas the remaining 992 NSTEMI patients underwent routine discharge. The primary end points of the study were the net adverse clinical events including major adverse cardiac or cerebral events or access site vascular/bleeding complications within 30 days. The differences between the two groups were analyzed after propensity score matching to reduce selection bias.

**Results:**

The incidence of crude 30-day net adverse events was numerically higher in the long-time discharge group at 11.6% (115/992) compared with 8.6% (37/432) in the short-time discharge group, although this difference was not significant (*P* = 0.09). This difference was mainly due to lesser radial access selected in the long-time discharge group (827/932, 83.4% vs. 387/432, 89.5%, *P* < 0.0005). After PS matching to balance the access difference, there was no significant difference in the incidence of the events mentioned above between two groups.

**Conclusions:**

If an NSTEMI patient undergoes PCI without any procedural or hospital complications, short-time discharge after successful PCI would be feasible and safe in selected NSTEMI patients.

## Background

Previous studies have demonstrated the safety and outcomes of short-term observation after elective percutaneous coronary intervention (PCI) with cautious patient selection [1–5], and the American College of Cardiology together with the Society for Cardiovascular Angiography and Interventions (ACC/SCAI) published a consensus document defining the length of stay following PCI [6]. However, these guidelines were only for cautiously selected patients, which restricted application of this consensus in real world practice. With approximately one million PCIs performed in China each year, more and more Non-ST elevation myocardial infarction (NSTEMI) patients are admitted for PCI treatment. However, these NSTEMI patients often undergo at least a 3 day hospitalization following an uncomplicated PCI due to worries that premature discharge can result in suboptimal care and increased potential for complications [7, 8]. With advances in technology and accumulating expertise over the past decades, especially with the more popular selection of radial access and various closure devices, PCI has become unequivocally safer and recovery easier. Therefore, cardiologists seek a short-term observation time following non-complicated PCI for high risk patients, such as NSTEMI patients. However, few studies have examined the impact of early hospital discharge on clinic outcomes in NSTEMI patients [9]. We examined the impact of a short time frame of observation (< 24 h) following non-complicated PCI on 30-day major adverse cardiac or cerebral events and net adverse clinical events in these NSTEMI patients.

## Methods

This retrospective study enrolled consecutive patients with NSTEMI diagnoses who were admitted to a cardiology unit at a tertiary hospital, Beijing Anzhen Hospital, in China, between May 2015 and May 2017. Demographic, clinical, echocardiographic, coronary angiographic and laboratory data at admission were gathered and recorded in a computerized database. All information was collected by trained medical staff. As a tertiary medical center and the biggest cardiovascular disease referral center among residents of the Beijing metropolitan area, approximately 10,000 PCI cases are performed at the Beijing Anzhen Hospital annually. From May 1, 2015, to May 1, 2017, a total of 2551 patients with NSTEMI diagnoses were admitted of whom 2108 (82.6%) were scheduled to undergo coronary angiography during hospitalization, and of these, 1916 (75.1%) underwent PCI procedures. The medical records and details of the PCI procedures were individually reviewed. Three hundred twenty-one cases of NSTEMI patients without hemodynamic stability or electrocardiology stability who required urgent PCI treatment or left ventricular supports were excluded. Of the remaining 1595 patients, 1424 PCI procedures met the non-complicated PCI criteria (criteria shown in Table 1). Four hundred thirty-two patients were discharged early (< 24 h after PCI procedures), and the remaining 992 NSTEMI patients were discharge after a routine period of time (2–3 days after procedures). This investigation was approved by our Institutional Review Board.

**Table 1:** Criteria for non-complicated PCI

No access site major bleeding	
No threatened vessel closure	
No prolonged “no reflow”	
No periprocedural hemodynamic instability	
No sustained ventricular or atrial arrhythmia	
No persistent dissection	
No compromised side branch flow	
No artery perforation	
No persistent or recurrent chest pain	
No ECG changes	

### Procedures

Percutaneous coronary intervention was performed using 6- to 7-F guiding catheters. Radial approach was used as the default, and femoral access was used in patients who were not suitable for radial approach. All patients were required to have received adequate oral antiplatelet therapy. Adequate pretreatment with P2Y12 inhibition was defined as chronic clopidogrel use or recent clopidogrel treatment with a 600 mg oral dose 2 h or more before the procedure or a 300 mg dose 6 h or more before the procedure; 180 mg ticagrelor could be used as a substitute for clopidogrel. For patients with chronic aspirin use, 100 mg aspirin was required. Otherwise, a 300 mg loading dose was required 6 h before the procedure.

According to the institutional protocol, activated clotting time was not routinely evaluated. For planned PCI procedures, 100 IU /kg heparin was given through the arterial sheath. For ad hoc PCI procedures, during the angiography, a single dose of 5000 IU heparin was given after insertion of the arterial sheath, and additional dose of heparin was given up to a total dose of 100 IU /kg.

The transradial arterial sheath was removed in the catheterization laboratory at the end of the procedure, and an occlusive tourniquet (TR Band, Terumo, Japan) was applied at the puncture site for at least 4 h until hemostasis was achieved. Closure devices were used in all suitable transfemoral access patients, and these included Perclose (Abbott Laboratory, California) and AngioSeal (St. Jude Medical, Minnesota). Hemostasis was then achieved by manual compression for the remaining patients.

After PCI, all patients underwent 12-lead electrocardiogram (ECG) analysis following PCI and before discharge. Routine enzymes were not systematically collected post-procedure if the procedures appeared uncomplicated. For patients who suffered unstable clinic symptoms such as chest pain, heart failure or ECG changes due to the PCI procedure, extended clinical observation, cardiac monitoring, or additional treatment were arranged as needed. The remaining patients without complications underwent hydration treatment and were observed for 12–18 h without cardiac monitoring. Patients who underwent transradial PCI were ambulated immediately after the procedure, and the transfemoral patients were ambulated after 4 to 6 h of bed rest if a closure device was used or after 12 to 18 h if the sheath was manually removed.

If the patients were free from symptoms, absence of electrocardiogram changes and puncture site abnormalities during post-procedure evaluation, when were they discharge on the operator’s discretion. If they discharge on the next day (< 24 h), they were included in the short-time discharge group. The remaining patients discharge on the 2–3 days after procedure and were included in the long-time discharge group.

According to the institutional protocol, patients were interviewed by telephone at 24 h and 3 days after discharge, and those who reported symptoms were referred to their own cardiologist. Thereafter, all patients were scheduled for an office visit with the cardiologist at 30 days. Post-interventional therapy included 100 mg/day of aspirin and 75 mg/day of clopidogrel or ticagrelor 90 mg twice/day at least for 12 months.

### Study outcomes

The end points of the study were the net adverse clinical events including major adverse cardiac and cerebral events or access site vascular/bleeding complications at 30 days. Major adverse cardiac and cerebral events were defined as cardiac death, myocardial infarction, stent thrombosis, stroke, coronary artery bypass grafting, and repeat PCI. Thrombolysis in Myocardial Infarction criteria were used to define bleeding. The vascular complications was defined as large hematoma, pseudoaneurysm, arteriovenous fistula, or any closure device–related complications such as abrupt closure, dissection, or thrombosis requiring re-admittance for invasive vascular intervention or surgery.

### Statistical analysis

Differences between categorical variables were tested using the chi-square test, and differences between continuous variables were tested using the *t*-test. We constructed a separate propensity score matching model for the short-time discharge and long-time discharge groups. This was to adjust for potential baseline differences between the groups. First, a logistic regression model was performed for clinical characteristics, angiography characteristics, procedure type, and admission type to calculate a propensity score for each individual patient. We then matched all patients using a 1:1 scheme without replacement and the nearest number matching method with caliper of 0.01. The Han-sen and Bowers balance test *p* value was 1.000, indicating good covariate balance. Survival rates for the short-time and long-time discharge groups were calculated using the Kaplan–Meier estimator and were compared using the log-rank statistic. Statistical significance was set at 0.05 (95%). Statistical analyses were performed using SAS 9.1 (SAS Institute, Inc., Cary, NC).

## Results

### Patient and procedure characteristics

The clinical characteristics of the patients are shown in Table 2, and compared with the long-time discharge group, there were fewer current smokers (38.9% vs. 31.8%, *P* = 0.004) in the short-time discharge group. Moreover, the left ventricular ejection fraction (LVEF) was lower in the short-time discharge groups (43 ± 11% vs. 45 ± 13%, *P* = 0.01), and there were more patients with LVEF less than 40% in the short-term group (21.9% vs. 19.8%, *P* = 0.03). B-type natriuretic peptide (BNP) and Troponin I (TnI) levels were greater in the short-time discharge group compared with the long-time discharge group (232 ± 24 pg/mL vs. 268 ± 33 pg/mL, *P* < 0.0005 and 16.45 ± 1.52 vs. 15.58 ± 1.46, *P* < 0.0005, respectively). There were 23.6% (102/432) patients underwent pre-treated with ticagrelor in short-time discharge group, which was similar as 24.9% (247/992) in long-time discharge group. At discharge, all the patients in both group accepted dual antiplatelet therapy (DAPT). The 186(43.1%) patients in short-time discharge group and 407(41.0%) patients in long time group accepted ticagrelor respectively, and remaining patients accepted clopidogrel, no significant difference were found between both groups. Radial access was a more popular choice in the short-time discharge group than the long-time discharge group (87.3% vs. 83.4%, *P* < 0.0005). Multiple stents (stents ≥2) were deployed more frequently in the short-time discharge group compared with the long-time discharge group (39% versus 36%, *P =* 0.01). After propensity score matching, these characteristics were well balance between both groups (shown in Table 3).

**Table 2:** Baseline clinical characteristics for the studied population before matching

Characteristics	Short-time group (*n* = 432)	Long-time group (*n* = 992)	*P* value
Age (years)	68 ± 11	67 ± 13	0.16
Male sex (*n*,%)	280 (64.7%)	653 (65.8%)	0.72
Diabetes mellitus (*n*,%)	172 (39.7%)	404 (40.7%)	0.77
Hypertension (*n*,%)	270 (62.4%)	605 (61.0%)	0.59
Current smoker (*n*,%)	137 (31.8%)	386 (38.9%)	0.04
Hypercholesterolemia (*n*,%)	270 (62.4%)	659 (64.4%)	0.16
Previous MI (*n*, %)	30 (6.9%)	62 (6.3%)	0.64
Previous PCI (*n*, %)	52 (12.1%)	116 (11.7%)	0.86
Prior coronary bypass surgery (*n*, %)	5 (1.2%)	8 (0.8%)	0.55
Renal insufficiency (*n*, %)	27 (6.3%)	62 (6.6%)	1.0
Left ventricular ejection fraction (%)	43 ± 11	45 ± 13	0.01
EF < 40% (*n*, %)	38 (21.9%)	54 (19.8%)	0.03
Peak troponin I (ng/ml)	16.45 ± 1.52	15.58 ± 1.46	< 0.0005
hs-CRP (mg/L)	22.8 ± 3.3	23.4 ± 3.6	< 0.0005
BNP (pg/mL)	232 ± 24	268 ± 33	< 0.0005
Radial Access (*n*, %)	387 (89.5%)	827 (83.4%)	< 0.0005
Targeted vessel (*n*, %)			
LM	8 (1.9%)	21 (2.1%)	0.94
LAD	145 (33.6%)	351 (35.4%)	
LCX	108 (25.0%)	249 (25.1%)	
RCA	170 (39.3%)	369 (37.2%)	
Saphenous vein graft	1 (0.2%)	2 (0.2%)	
Lesion type (n, %)			
A	81 (18.7%)	186 (18.8%)	0.91
B1	52 (12.1%)	128 (12.9%)	
B2	167 (38.7%)	365 (36.7%)	
C	132 (30.5%)	313 (31.6%)	
Multivessel disease (*n*, %)	91 (21.1%)	218 (22.0%)	0.73
Multivessel intervention (*n*, %)	83 (19.2%)	188 (18.9%)	1.00
Multilesion intervention (*n*, %)	124 (28.7%)	287 (28.9%)	0.94
Stent length (mm)	24.1 ± 7.3	19.7 ± 6.8	
Stents ≥2 (*n*, %)	188 (43.5%)	360 (36.3%)	0.01
Side-branch involvement (*n*, %)	99 (23.0%)	249 (25.1%)	0.38
Calcification lesion (*n*, %)	121 (28%)	272 (27.4%)	0.85
Total occlusion lesion (*n*, %)	96 (22.2%)	229 (23.1%)	0.78
Usage of ticagrelor (*n*, %)	186 (43.1%)	407 (41.0%)	0.75
Usage of glycoprotein IIb/IIIa inhibitors (*n*, %)	135 (31.1%)	317 (32.0%)	0.80

Abbreviations: *MI* Myocardial infarction, *PCI* Percutaneous coronary intervention, hs-CRP, high-sensitivity C-reactive protein, *BNP* B-type natriuretic peptide, *LM* Left main, *LAD* Left anterior descending; *LCX* Left circumflex, *RCA* Right coronary artery

**Table 3:** Baseline clinical characteristics for the studied population after matching

Characteristics	Short-time group (*n* = 432)	Long-time group (*n* = 432)	*P* value
Age (years)	68 ± 11	67 ± 13	0.22
Male sex (*n*,%)	280 (64.7%)	276 (63.9%)	0.89
Diabetes mellitus (*n*,%)	172 (39.7%)	174 (40.3%)	0.94
Hypertension (*n*,%)	270 (62.4%)	272 (63.0%)	0.95
Current smoker (*n*,%)	137 (31.8%)	135 (31.3%)	0.94
Hypercholesterolemia (*n*,%)	270 (62.4%)	276 (63.8%)	0.94
Previous MI (*n*, %)	30 (6.9%)	28 (6.5%)	0.89
Previous PCI (*n*, %)	52 (12.1%)	55 (12.7%)	0.84
Prior coronary bypass surgery (*n*, %)	5 (1.2%)	4 (0.9%)	1.00
Renal insufficiency (*n*, %)	27 (6.3%)	30 (6.9%)	0.78
Left ventricular ejection fraction (%)	43 ± 11	42 ± 8	0.13
EF < 40% (*n*, %)	38 (21.9%)	38 (21.9%)	1.00
Peak troponin I (ng/ml)	16.45 ± 1.52	16.28 ± 1.82	0.14
hs-CRP (mg/L)	22.8 ± 3.3	22.9 ± 3.1	0.64
BNP (pg/mL)	232 ± 24	235 ± 23	0.06
Radial Access (*n*, %)	387 (89.5%)	376 (87.0%)	0.29
Targeted vessel (*n*, %)			
LM	8 (1.9%)	9 (2.1%)	0.99
LAD	145 (33.6%)	143 (33.1%)	
LCX	108 (25.0%)	110 (25.0%)	
RCA	170 (39.3%)	169 (39.1%)	
Saphenous vein graft	1 (0.2%)	1 (0.2%)	
Lesion type (*n*, %)			
A	81 (18.7%)	83 (19.3%)	0.99
B1	52 (12.1%)	55 (12.7%)	
B2	167 (38.7%)	164 (37.9%)	
C	132 (30.5%)	130 (30.1%)	
Multivessel disease (*n*, %)	91 (21.1%)	93 (21.5%)	0.93
Multivessel intervention (*n*, %)	83 (19.2%)	78 (18.1%)	0.73
Multilesion intervention (n, %)	124 (28.7%)	122 (28.2%)	0.94
Stent length (mm)	24.1 ± 7.3	24.2 ± 8.1	0.85
Stents ≥2(n, %)	188 (43.5%)	173 (40.0%)	0.33
Side-branch involvement (*n*, %)	99 (23.0%)	93 (21.5%)	0.94
Calcification lesion (*n*, %)	121 (28%)	118 (27.3%)	0.88
Total occlusion lesion (*n*, %)	96 (22.2%)	98 (22.7%)	0.94
Usage of ticagrelor (*n*, %)	186 (43.1%)	178 (41.2%)	0.78
Usage of glycoprotein IIb/IIIa inhibitors (*n*, %)	135 (31.1%)	138 (31.9%)	0.88

Abbreviations: *MI* Myocardial infarction, *PCI* Percutaneous coronary intervention, *hs-CRP* High-sensitivity C-reactive protein, *BNP* B-type natriuretic peptide, *LM* Left main, *LAD* Left anterior descending, *LCX* Left circumflex, *RCA* Right coronary artery

### Clinical outcomes

Three patients deaths occurred in the short-time discharge group, and 7 deaths occurred in the long-time discharge group (0.69% vs. 0.71%, *P* = 1.00) during the 30-day follow-up period. The total MACCE incidence was not significantly different in the short-time discharge group compared with the long-time discharge group (2.9% vs. 3.8%, *P* = 0.54). The incidences of 30-day cerebrovascular events (1.2% vs. 1.4%; *P* = 0.80), definite/ probable stent thrombosis (0.7% vs. 0.9%; *P* = 1.00), and major bleeding (1.6% vs. 2.0%, *P* = 0.68) were not different between the short-time and long-time groups (shown in Table 4).

**Table 4:** Thirty- day crude occurrence of net adverse clinic events

	Short-time group(*n* = 432)	Long-time group(*n* = 992)	Hazard Ratio (95% CI)	*P* value
MACCEs (*n*,%)	13 (3.0%)	38 (3.8%)	0.42–1.48	0.54
Death (*n*,%)	3 (0.69%)	7 (0.71%)	0.26–3.83	1.00
Myocardial infarction (*n*,%)	8 (1.9%)	29 (2.9%)	0.28–1.38	0.28
STEMI	2 (0.5%)	10 (1.0%)	0.09–2.09	0.53
NSTEMI	6 (1.4%)	19 (1.9%)	0.29–1.81	0.66
Definite/ Probable Stent thrombosis (*n*,%)	3 (0.7%)	9 (0.9%)	0.21–2.84	1.00
Stroke (*n*,%)	5 (1.2%)	14 (1.4%)	0.29–2.82	0.80
Bleeding and access related complications (*n*,%)	24 (5.6%)	77 (7.8%)	0.44–1.12	0.15
Major Bleeding	7 (1.6%)	20 (2.0%)	0.34–1.91	0.68
Arteriovenous fistula	2 (0.46%)	15 (1.50%)	0.07–1.33	0.11
False aneurysm	5 (1.2%)	18 (1.8%)	0.23–1.72	0.49
Hematoma	11 (2.5%)	29 (2.9%)	0.43–1.75	0.86
Net adverse clinic events (n,%)	37 (8.6%)	115 (11.6%)	0.49–1.05	0.09

Abbreviations: *MACCE*, Major adverse cardiac and cerebral events; *CI* Confidence interval, *STEMI* ST-elevation myocardial infarction; *NSTEMI* Non-ST-elevation myocardial infarction

**Fig. 1:**
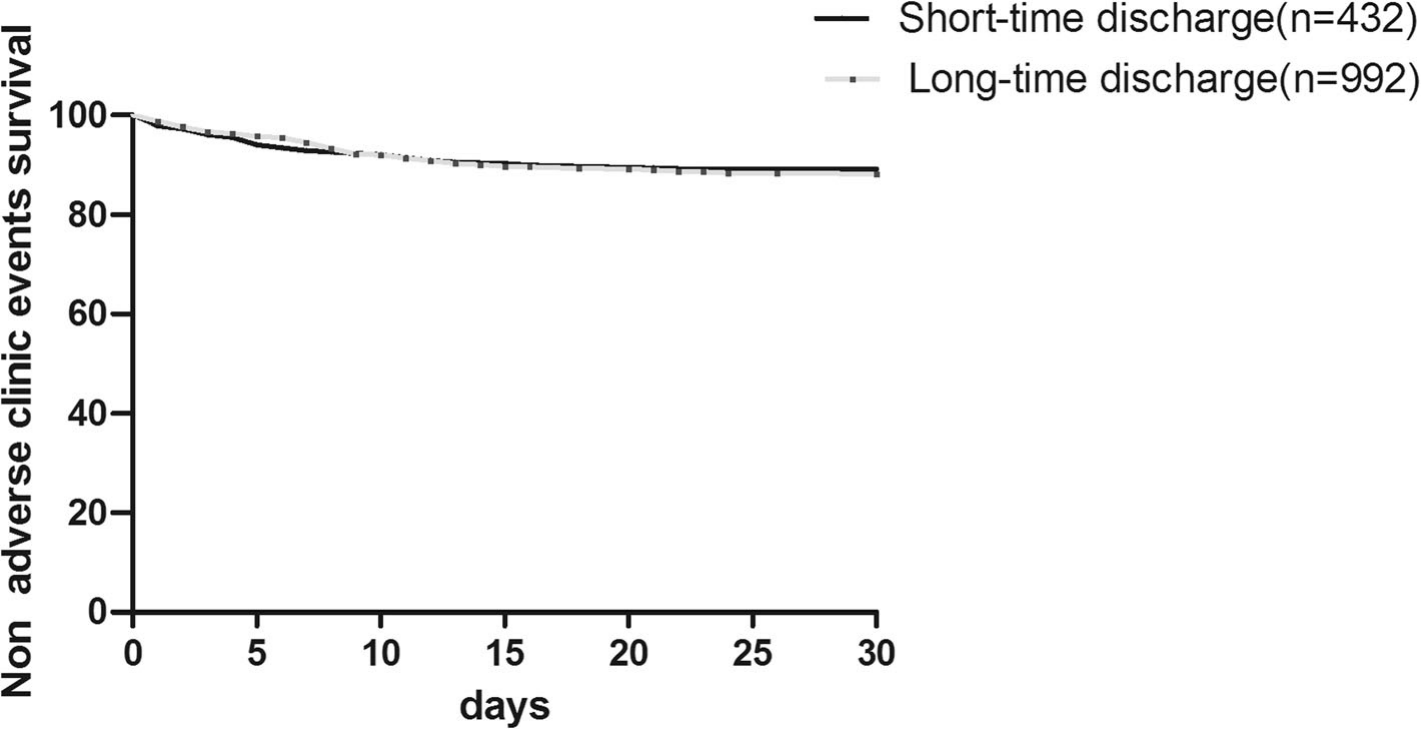
Kaplan–Meier survival curves for crude cumulative non-adverse clinic event survival in the short-time and long-time discharge groups before matching. Survival curves were compared using log-rank test, there was no significant difference between short-time discharge group and long-time discharge group (*P* = 0.69).Short-time discharge: short-time discharge group (*n* = 432), Long-time discharge: long time discharge group (*n* = 992)

The crude 30-day net adverse events incidence was higher in the long-time discharge group numerically at 11.6% (115/992) compared with 8.6% (37/432) in the short-time discharge group, although this difference was not significant (*P* = 0.09). This difference was mainly due to the higher incidences of access-related complications, such as bleeding, arteriovenous fistula, false aneurysm, and hematoma, which were higher numerically in the long-time discharge group compared with the short-time discharge group (7.8% vs. 5.6%, *P* = 0.15). The Kaplan–Meir non-adverse event survival curves revealed no significant difference (*P* = 0.69) between the short-time discharge and long-time discharge groups (Fig. 1).

**Fig. 2:**
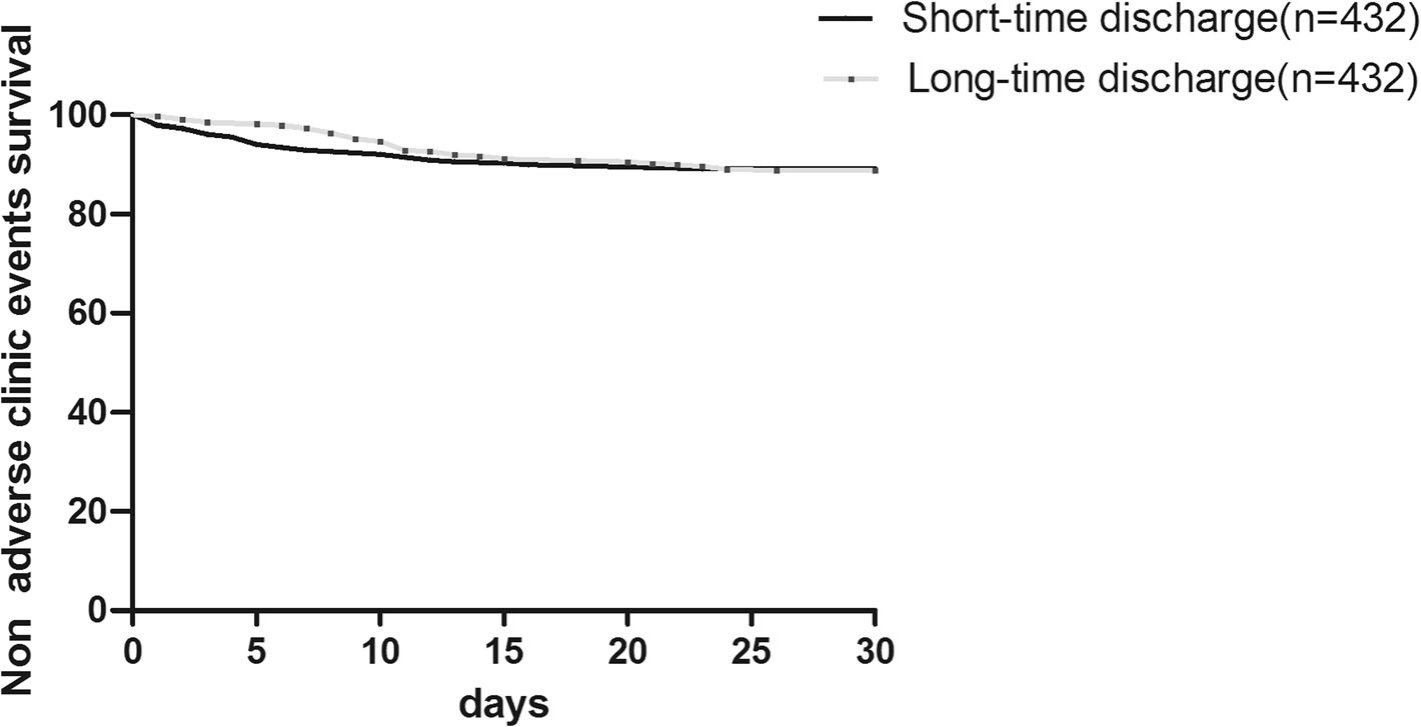
Kaplan–Meier survival curves for cumulative non-adverse clinic event survival in the short-time and long-time discharge groups after matching. Survival curves were compared using log-rank test, there was no significant difference between short-time discharge group and long-time discharge group (*P* = 0.97).Short-time discharge: short-time discharge group (*n* = 432), Long-time discharge: long time discharge group (*n* = 432)

The propensity score matching produced a well-balanced matched pair sample, balance with radial access selection between groups resulted in lower rates of bleeding and access-related complications (26/432,6.0%) in the long-time discharge group, and which was similar as the rates in the short-time group (24/432,5.6%, *P* = 0.88). After PS match, there was no significant difference in other events incidence mentioned above between two groups also (shown in Table 5). Figure 2 shows the Kaplan–Meir non-adverse event survival curves between the groups after PS matching (*P* = 0.97).

**Table 5:** Thirty-day Occurrence of net adverse clinic events after matching

	Short-time group (*n* = 432)	Long-time group (*n* = 432)	Hazard Ratio (95% CI)	*P* value
MACCEs (*n*,%)	13 (3.0%)	12 (2.8%)	0.49–2.41	1.00
Death (*n*,%)	3 (0.69%)	3 (0.69%)	0.20–4.92	1.00
Myocardial infarction (*n*,%)	8 (1.9%)	7 (1.6%)	0.41–3.20	1.00
STEMI	2 (0.5%)	3 (0.7%)	0.11–3.97	1.00
NSTEMI	6 (1.4%)	4 (0.9%)	0.43–5.28	0.75
Definite/ Probable Stent thrombosis (*n*,%)	3 (0.7%)	5 (1.2%)	0.17–3.37	0.73
Stroke (*n*,%)	5 (1.2%)	5 (1.2%)	0.29–3.43	1.00
Bleeding and access-related complications (*n*,%)	24 (5.6%)	26 (6.0%)	0.52–1.63	0.88
Major Bleeding	7 (1.6%)	8 (1.8%)	0.29–2.07	1.00
Arteriovenous fistula	2 (0.46%)	1 (0.23%)	0.18–21.9	1.00
False aneurysm	5 (1.2%)	4 (0.9%)	0.33–4.70	1.00
Hematoma	11 (2.5%)	12 (2.1%)	0.40–2.10	1.00
Net adverse clinic events (n,%)	37 (8.6%)	38 (8.8%)	0.60–1.56	1.00

Abbreviations: *MACCEs* Major adverse cardiac and cerebral events, *CI* Confidence interval, *STEMI* ST-elevation myocardial infarction, *NSTEMI* Non-ST-elevation myocardial infarction

## Discussion

To the best of our knowledge, the present study is the first study of short-time discharge after NSTEMI patients underwent successful PCI procedure. It demonstrates that short-time (< 24 h) discharge after non-complicated PCI did not lead to unattended cardiac events or to more access complications. This suggested that short-term discharge in selected NSTEMI patients is likely safe.

NSTEMI patients comprise approximately 70% of all cases of myocardial infarction, and their mortality is similar to that observed in patients with ST-segment elevation myocardial infarction after one year of acute events [10, 11]. Several clinical trials proved the efficacy and safety of PCI strategy for NSTEMI patients [12–14]. However, for this high-risk population, the longer observed time after PCI is mandatory, and whether the shorter length of stay (LOS) would adversely affect health outcomes of NSTEMI patients remains controversial. Few RCT studies have investigated the rational length of stay (LOS) after non-complicated PCI in NSTEMI patients. The EASY Trial [9] enrolled a modestly high-risk patients as 20% arrived to the procedure with abnormal troponin levels. If the procedure was successful without complications, this trial demonstrated non-inferiority to short-term discharge compared with long-term discharge. However, those patients enrolled into the EASY trail were limited in range, and whether this result could apply to the real-world spectrum of practice is impossible to determine. In contrast, in our study, patients with NSTEMI diagnoses were enrolled consecutively in the real world and with a limited number of exclusions, the patients had diverse demographics, comorbidities, and risk factors with complex coronary lesions. From our study, the 30-day major adverse cardiac cerebral events for the short-time discharge PCIs were low (3.0%) and comparable to those in the long-time discharge group (3.8%). The lower incidence of major adverse cardiac cerebral events in the short–time discharge group demonstrated that the longer observation time after non-complicated PCI in NSTEMI patients is not necessary, and our study in a diverse patient population supports that short-time discharge after successful PCI can be safely performed in the real world.

As high-risk patients, NSTEMI patients have extreme variations in clinical and angiographic characteristics, and multiple risk models dominated by patient demographics, underlying disease, and angiographic characteristics have evolved to determine the potential risk of NSTEMI patients [15, 16]. However, if the PCI procedure was uncomplicated and the stented result was satisfactory, the short-term outcome is excellent with little influence from underlying patient characteristics. In the present study, many patients with complex angiographic characteristics (including left main lesion, bifurcation lesion, and total occlusion lesion) and serious clinic condition (including very old patients and those with diabetes mellitus, low LVEF and renal insufficiency) were discharged early after successful PCI procedure, and these factors were not associated with poorer outcomes. These results support that residual risk of high-risk patients after PCI treatment shift to be dependent on the mechanical success of the procedure [17]. Therefore, as high-risk patients, NSTEMI patients after successful PCI treatment can be discharged early as in elective PCI patients. However, the rates of early stent thrombosis of acute coronary disease are higher compared with the patients who underwent selective PCI treatment; 5 cases of early stent thrombosis occurred 12–18 h after procedure in our study. Furthermore, contrast-induced nephropathy prevention is also extremely important for ACS patients, and adequate hydration treatment (> 12 h) is the most important method to prevent contrast-induced nephropathy. Third, many bleeding complications occurred several hours after ambulation, and timely detection and treatment of those complications within the observed time would prevent readmission and serious clinical consequences. Through the facts mentioned above, it is suggested that overnight(< 24 h) discharge after successful PCI for NSTEMI patients would be a safe strategy, whereas same day discharge after PCI for NSTEMI patients as reported should be strictly limited in real world practice [18].

Post procedure bleeding complications are as severe as ischemia complications, and greater anticoagulation and antiplatelet therapy for NSTEMI patients causes more bleeding complications and access site complications. The transradial approach is the preferred approach for NSTEMI patients and clearly reduced the risk of access instability and bleeding [19, 20]. In the present study, the crude rates of bleeding and other access-related complications were higher numerically in the long-time discharge group mainly due to less frequent selection of radial access sites (84.7% in the long-time group vs. 89.5%, *P* < 0.005). Although the closure devices were used routinely in femoral approach patients in this study, those devices enabled early ambulation but did not reduce the rates of access-related complications. After PS matching, balance with radial access selection between groups resulted in similar rates of bleeding and access-related complications in the both groups (26/432, 6.0% vs.24/432,5.6%, *P* = 0.88). These results support that the uptake of radial access for patients undergoing PCI can reduce the average length of stay for NSTEMI patients due to fewer access-related complications and shorter monitoring time requirements.

### Limitations

This study does have some significant limitations. This investigation was an observational study conducted at a single center, and the patient sample size was limited. This study was also not a randomized series that can control for variability between groups, even after data adjustment, and the results could be biased by potentially important parameters that were not available in the study. The cases in this study were conducted in a very large PCI volume center with experienced staff, therefore, the present results cannot be extrapolated to settings with less-experienced operators and/or to low volume centers. Finally, only 30-day outcomes were analyzed in the present study, and whether long-term differences in outcome are affected by early discharge is also left unanswered.

## Conclusions

These findings from our study demonstrate that early (< 24 h) discharge does not lead to additional complications compared with a routine long-time stay for NSTEMI patients, and which imply that short-time discharge after successful PCI is feasible and safe in the selected NSTEMI patients.

## Abbreviations

BNP: B-type natriuretic peptide
DAPT: Dual antiplatelet therapy
LOS: Length of stay
LVEF: Left ventricular ejection fraction
MACCEs: Major adverse cardiovascular and cerebral events
MI: Myocardial infarction
NSTEMI: Non-ST elevation myocardial infarction
PCI: Percutaneous coronary intervention
TnI: Troponin I
